# Open or closed: Experience of head and neck radiotherapy masks – A mixed‐methods study

**DOI:** 10.1002/jmrs.825

**Published:** 2024-09-27

**Authors:** Erik Lundin, Sofia Axelsson, Emma Ohlsson‐Nevo

**Affiliations:** ^1^ Department of Oncology, Faculty of Medicine and Health Örebro University Örebro Sweden; ^2^ Faculty of Medicine and Health Örebro University Örebro Sweden; ^3^ Faculty of Medicine and Health, University Health Care Research Center Örebro University Örebro Sweden

**Keywords:** Anxiety disorders, head and neck neoplasms, quality of life, radiation equipment and supplies, radiotherapy

## Abstract

**Introduction:**

In radiotherapy for head and neck cancer, a mask is used to immobilise the head and shoulders. An open mask that does not cover the face is expected to cause less anxiety, but there is need to further investigate the patients' experience of open versus closed masks. Therefore, the aim of this study is to evaluate patient preferences for open or closed masks and whether an open mask can reduce discomfort and anxiety for patients.

**Methods:**

Twenty participants were treated in alternating weeks using open and closed masks. Their distress was evaluated through semi‐structured interviews and patient‐reported outcome measures.

**Results:**

When using the open mask, it took longer to position the patient correctly. The closed mask felt more confining and could induce a sense of claustrophobia. Participants employed both internal and external strategies to cope with the stressful situation. The Hospital Anxiety and Depression Scale (HADS) showed a significant reduction in anxiety over time during the treatment period, but no significant difference between the masks. When participants chose which mask to use for the final treatments, 12 chose the open mask, while 8 chose the closed mask. In addition to the 20 analysed participants, two participants withdrew from the study because they could only tolerate the open mask, one due to anxiety and the other due to swelling.

**Conclusions:**

The open mask seems to provide a less confined experience but may lead to greater difficulties in achieving the correct treatment position. While both masks can be viable options for most patients, some cannot tolerate closed masks but do tolerate open masks.

## Graphical abstract

Patients with head and neck cancer received radiotherapy alternately with an open and a closed mask. The closed mask was perceived as more confining and restrictive, but it was also considered to make it easier to quickly achieve the correct position, while the open mask was perceived as less stable, although it reduced the feeling of claustrophobia for some participants. Most participants did not have a strong preference, and no difference between the masks could be detected using the HADS.

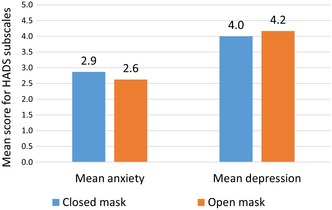

## Introduction

Radiotherapy for the head and neck region requires effective positioning and immobilisation of the head, neck and shoulders, which is typically achieved with X‐ray imaging and the use of a thermoplastic mask individually manufactured to fit the patient.^1^ When applied, the mask severely restricts movement, covering most of the patient's face.

The mask can cause discomfort, anxiety, feelings of panic, and a sense of vulnerability and exposure,^2^, ^3^ affecting the ability to undergo treatment.^4^, ^5^, ^6^ For most patients, anxiety subsides during the course of treatment (72%), but for some, symptoms persist (22%) or worsen (6%) over time.^7^ Patients with psychiatric medication, claustrophobia, phobia of being covered or restrained, or a history of panic attacks are at increased risk of anxiety problems.^8^ To counteract this, various support measures from the staff, such as increased communication and visual information to increase patient preparedness, as well as coping strategies such as relaxation, self‐distraction, breath regulation and meditation, have been proposed.^4^, ^9^, ^10^, ^11^

A new type of open mask, which leaves a large opening for the face, is presumed to reduce discomfort and be easier for people with claustrophobic disorders to tolerate.^12^, ^13^ The open mask also allows for patient positioning and monitoring with laser‐based surface scanning. When position changes are detected, the treatment is interrupted and resumed after correction.^14^

Two small prospective, randomised studies report good results regarding both clinical safety of positioning and patient experience with open masks.^12^, ^15^ However, we are not aware of any reported studies where patients have alternated between open and closed masks to evaluate differences in comfort.

In this study, participants alternately used open and closed masks, allowing them to serve as their own controls and thus providing greater statistical power when comparing patient‐reported outcome measures. Participants directly experienced the differences between the two masks, which informed their decision on which mask to use during the final four treatments. They were also able to express their thoughts on the pros and cons of both masks during interviews.

The aim of this study was to evaluate patient preferences for open or closed masks and to determine whether the open mask can reduce discomfort and anxiety. This study will contribute to the understanding of patient experiences with masks during radiotherapy and support the consideration of alternative options for routine clinical care in radiotherapy for the head and neck region.

## Methods

### Design

A convergent mixed‐method research design was employed, where both quantitative and qualitative data were collected, analysed and compared to determine whether the data confirmed or disconfirm each other.^16^

### Sample

The study protocol specified that 20 consecutive patients scheduled for treatment with 68Gy in 34 fractions for head and neck tumours at the radiotherapy department at Örebro University would be included. The number of participants was determined based on power calculations for a study planned to investigate the effectiveness of the masks in immobilising and positioning patients, and this study was then planned using the same study sample without any additional power calculation being conducted. Treatment for 34 fractions has long been a standard curative treatment in this situation, and it was considered sufficient for participants to gain experience with both masks and make a well‐informed comparison. Exclusion criteria were serious concomitant morbidity, pregnancy or participation in any other radiotherapy study. Inclusion was interrupted several times due to the ongoing COVID‐19 pandemic, and therefore, completion of the study took longer than planned.

Oral and written information was provided, and written informed consent was obtained from all participants. The study received ethical approval from the Swedish Ethics Review Board (D.nr 2019‐00249).

### Treatment procedures

**Figure 1:**
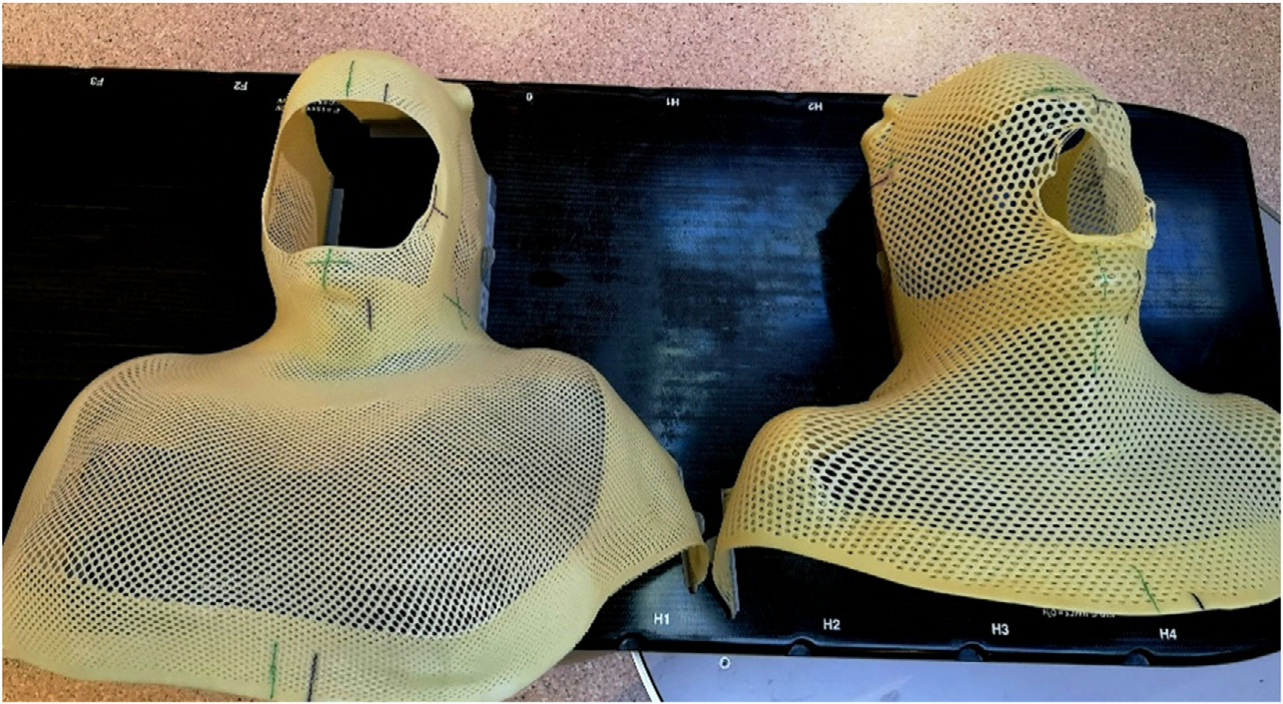
Open mask on the left (Orfit 5‐point open‐face hybrid mask) and closed mask on the right (Orfit 5‐point hybrid mask) (Orfit Industries, Belgium).

A conventional closed mask and an open mask, both with five‐point fixation, were produced for each participant (Fig. 1). The same neck support was used for both masks. Two computerised tomography (CT) scans were performed with the conventional closed mask and open mask. Both CT scans were imported into Aria/Eclipse (Varian Medical Systems, Palo Alto, CA, USA).

A radiation oncologist defined the target volumes and organs at risk. Deformable image registration was used to ensure that target structures and organs at risk were as similar as possible in the structure sets for both CT scans. Dose planning was performed on both CT scans, aiming to ensure equivalent treatment with the two masks.

Randomisation determined which mask the participant used during the first week. The masks were then switched every fifth treatment. Before the last week of treatment, participants were free to choose which mask to use for the final 4 days.

Participants using the closed mask were initially positioned by aligning the markings on the mask with laser lines, and the position was further adjusted using orthogonal kV images. For open‐mask treatment, initial positioning was performed with the AlignRT® surface scanning system, which uses facial structures for positioning, with the participant's outline from the dose planning CT used as a reference. The position was then adjusted using orthogonal kV images.

### Quantitative data collection

Patient‐reported outcome measures with the Hospital Anxiety and Depression Scale (HADS) were recorded at the start of treatment and before the first treatment after each mask change. HADS consists of two subscales measuring symptoms of depression and anxiety during the previous week. Scores of 8 or below indicate the absence of symptoms, scores above 8 indicate a possible case, and scores above 11 indicate a likely case of anxiety or depression.^17^, ^18^

After the final treatment before each mask change, participants completed two questionnaires:

The European Organisation for Research and Treatment of Cancer (EORTC) core questionnaire QLQ‐C30, an instrument for assessing general health‐related quality of life during the past week for cancer patients.^19^

The EORTC module for head and neck cancer, QLQ‐H&N43, a supplementary questionnaire to QLQ‐C30 that addresses issues specific to head and neck cancer patients.^20^

### Statistical analysis

Changes in the mean value of both HADS sub‐scores were analysed when using the two different masks, as well as changes over time during radiotherapy for both HADS sub‐scores and all separate domains of QLQ‐C30 and QLQ‐H&N43. The changes in the mean values were tested for statistical significance with paired‐sample *T*‐test, double‐sided.

Regarding participants' choice of mask for the final four treatments, we used the null hypothesis that both masks would be chosen equally often (i.e. 50% of participants would prefer each). We then tested whether the null hypothesis could be rejected using a one‐sample binomial test. IBM SPSS v.28 software (IBM, Armonk, NY, USA) was used for all statistical calculations.

### Qualitative data collection

Individual interviews were conducted within 2 weeks after the end of radiotherapy by one of the authors, an experienced nurse at the radiotherapy department who was not involved in treating the study participants. Participants decided the time, day and place of the interview. Data were collected through semi‐structured interviews with open‐ended questions. The interview guide questions are shown in Table 1. Interviews were digitally recorded and later transcribed verbatim.

**Table 1:** Questions in the interview guide.

Opening question	How did you find the radiotherapy treatment as a whole?
Follow‐up questions	How did you feel about the open mask?
	How did you feel about the closed mask?
	How did you feel about the red light?
	Please describe how you felt the day before your treatment.
Probing questions	Can you elaborate?
	What do you mean by that?
	Tell me more

These questions were originally developed in Swedish, and what is presented here is the closest translation into English.

The transcribed text was analysed using conventional content analysis as described by Hsieh et al.^21^ To gain a sense of the whole and check for accuracy, the text was read while simultaneously listening to the recordings. Each transcript was divided into meaning units and labelled with a code. All codes were compared, and categories were identified based on differences and similarities. The authors held regular discussions throughout the analysis. Two of the authors have extensive experience with radiation treatment, and one author is a senior researcher with expertise in conducting surveys and descriptive qualitative methods. This resulted in a richness and depth in the imaginative interpretation and possible variations during data analysis and interpretation.

## Results

Twenty‐two participants were enrolled, and 20 completed the study. One participant had to discontinue participation due to swelling and one due to claustrophobia. Both could tolerate the open mask but not the closed one. Baseline data are presented in Table 2.

**Table 2:** Characteristics of study participants.

	Mean	SD
Age (years) range 47–73	62.5	8

	***n***	**%**
Gender		
Male	16	80
Female	4	20
Marital status		
Unmarried	1	5
Married	17	85
Widow/Widower	1	5
Divorced	1	5
Education level		
Elementary school	3	15
High school	7	35
College/University	8	40
Other	2	10
Employment		
Employed	7	35
Self employed	3	15
Retired	10	50
Diagnosis		
Oropharyngeal cancer	18	90
Other	2	10
Concurrent chemotherapy		
Yes	5	25
No	15	75

Interviews were conducted with 16 participants. The interviews were concluded when similar experiences were being shared. All 20 participants completed HADS, QLQ‐C30 and QLQ‐H&N43 questionnaires. In total, there were less than 2% missing values in these instruments. Missing values occurred when participants did not complete all data points or when staff failed to distribute the forms on one occasion.

Three themes emerged from the interviews:

### Theme 1: The patient experience of producing the mask

The process of preparing the individual masks was described in various ways: some found it interesting and exciting, while others found it unpleasant, troublesome and claustrophobic, with strong feelings of loneliness, anxiety, suffocation and even thoughts of dying. Some participants found the mask warm and had no trouble lying still while it hardened, while others experienced discomfort, describing a crawling sensation across their face. Knowing that the actual radiotherapy session would last a shorter time was a comforting factor.

It felt unpleasant when the mask dried and shrank, it felt like my entire face was crawling and I thought that there was something wrong. (pat. 9).

No problem lying still and letting the mask harden. It was just a matter of relaxing and leaving myself in the hands of the professionals. (pat. 10).

### Theme 2: The patient experience of wearing the mask during treatment

The actual radiotherapy treatment was perceived in different ways, ranging from a necessary evil to something almost impossible to endure, but also as exciting, cool and interesting. The mask elicited a variety of feelings, from relaxation to feelings of claustrophobia. Wearing the mask was described as easy, interesting or something one needed to get used to. Some participants fell asleep during treatment, while others had a mental or intellectual struggle being trapped in the mask.

Some participants felt terrified of the thought of having to move or swallow, as they were not allowed to. They had to concentrate and focus on being calm and still.

I don't dare to move I don't dare to swallow. (pat. 1).

Both masks were described as firm, which was generally perceived positively, as participants felt it enabled them to lie still and ensured the treatment was accurate. As treatment progressed, some participants found it easier to handle being immobilised with the mask, whether it was an open or closed one, reducing their reliance on internal and external coping strategies.

Masks are great in terms of being able to lie still, and the radiation is on target. (pat. 7).

Based on their experiences with both mask types during radiotherapy, several participants speculated about which mask would be more suitable for other individuals. They reasoned about whether the open mask would be better for those with claustrophobia.

I was thinking about my own experience with claustrophobia and how things might be for other people. Which mask should be chosen. (pat. 3).

A common experience with the open mask was that it was more difficult to get the head into the perfect position compared to the closed mask. The participant had to adjust the chin, nose and head and the staff had to spend time to get it right.

The open mask was perceived as less stable and weaker than the closed mask, but it provided a greater sense of freedom, allowing participants to look around and engage with their surroundings. Participants could choose to close their eyes, watch the ceiling or observe the treatment machine moving around their head. The open mask also offered better breathing opportunities and was considered better for those with coughing issues.

Some participants experienced reduced feelings of claustrophobia with the open mask, but the sensation of confinement could still persist, accompanied by discomfort from being restrained.

Feeling that you are not secured as well with the open one. That the staff have to adjust your head more. The feeling of wanting to be helpful and lie just so in order to ensure that the treatment will be right. (pat. 7).

The open one is freeing. You get more involved with your surroundings and the staff around you. (pat. 8).

The red laser light used to position the participant in the open mask was not perceived as a concern or as disturbing, although the light could be perceived as a bit strong.

The closed mask was perceived as more stable and better at achieving the correct treatment position immediately, which was smoother for both participants and staff. There was no need for the participants to help adjust the position and that could help the participant to be relaxed. However, the closed mask was also described as more confining, causing feelings of entrapment, as the participant was stuck. The mask could pinch and cause an unpleasant pressure as the mask was squeezed over the face. The closed mask covered the eyes, which could be bothersome and unpleasant for some participants, as it prevented them from keeping their eyes open during treatment. On the other hand, some participants found it easier to relax in the closed mask because their eyes were closed. Others experienced severe anxiety and panic attacks, requiring medication to complete the planned session with the closed mask.

I like the closed one better because I was more relaxed in it. Felt that I was better situated. (pat. 9).

A perception of being trapped and a feeling of panic. My body was shuddering. (pat. 3).

### Theme 3: Patient strategies to overcome the discomfort

Being mentally prepared and knowing what to expect of the treatment sessions as well as one's personal reaction were described as helpful. Forcing oneself to think of something else, about nice things or what to do afterwards, or putting oneself in another place mentally, were found to be helpful strategies to relax and overcome. Participants with experience of mindfulness or prophylactic breathing were well helped by practising this during treatment. Breathing exercises such as inhaling through the mouth and exhaling through the nose were described as being helpful. Counting the heartbeats was another active way to be distracted from the situation.

Being in the mask was doable. I thought about this and that, about just about anything, like what I was going to do later on. (pat. 2).

If you take a breath through your mouth and hold it for 10 seconds and then let it out through your nose, and you do that several times. And then you feel how the tension in your body relaxes. (pat. 16).

Besides sedative drugs, a common strategy was to arrive early for treatment to sit in the waiting room, prepare by swallowing, drinking water, calming down and making sure to cough before the treatment so they would not have to cough during the treatment. The participants described how they experienced support from the nurses and how important that support was to be able to undergo the treatment. The knowledge that help was available strengthened the participants' own coping strategies.

The staff's actions when they lay me up on the bench and how they take care of me make me feel comfortable and safe. (pat. 11).

### Quantitative measurements of anxiety and depression

At the start of treatment, four participants (20%) reported scores on the HADS anxiety subscale indicating possible anxiety problems. For one participant, this persisted throughout the study, while the other three reported normal scores in subsequent measurements. The mean value of the HADS anxiety subscale for all participants decreased in the first week and then remained stable. The change from the first to the last measurement was statistically significant (*P* < 0.001).

**Figure 2:**
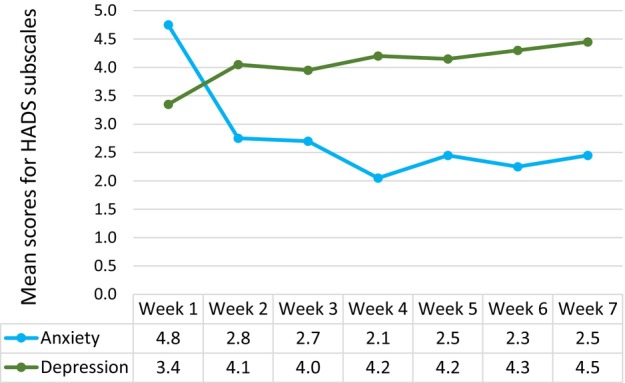
Patient‐reported Hospital Anxiety and Depression Scale (HADS) scores from week 1 to week 7. Statistical significance for change from first to last measurement (paired‐sample *T*‐test, double‐sided): anxiety, *P* < 0.001; depression, *P* = 0.132.

At the start of treatment, three participants (15%) reported scores indicating possible depression, with two (10%) scoring above 10, suggesting probable depression. Participants who reported high depression scores at the start generally continued to do so. The mean value for the HADS depression subscale increased slightly during the study period, though the change was not statistically significant. Development over time for both HADS subscales is shown in Figure 2.

**Figure 3:**
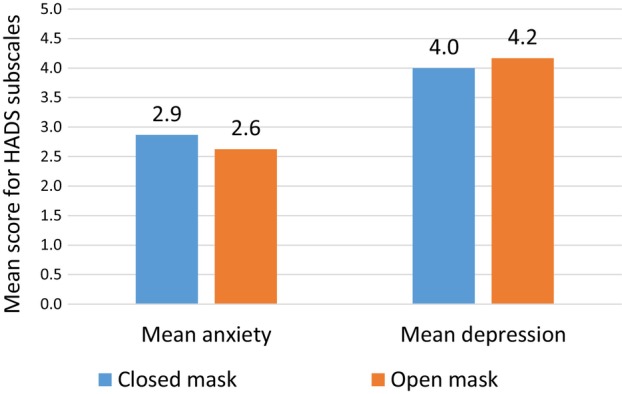
Hospital Anxiety and Depression Scale (HADS) scores with closed mask and open mask at the start of treatment. Statistical significance for difference between closed and open masks (paired‐sample *T*‐test, double‐sided): mean anxiety, *P* = 0.153; mean depression, *P* = 0.170.

Mean scores for the HADS anxiety and depression subscales did not show a statistically significant difference between weeks with open and closed masks. Mean scores are presented in Figure 3.

### Quantitative quality‐of‐life measurements

**Figure 4:**
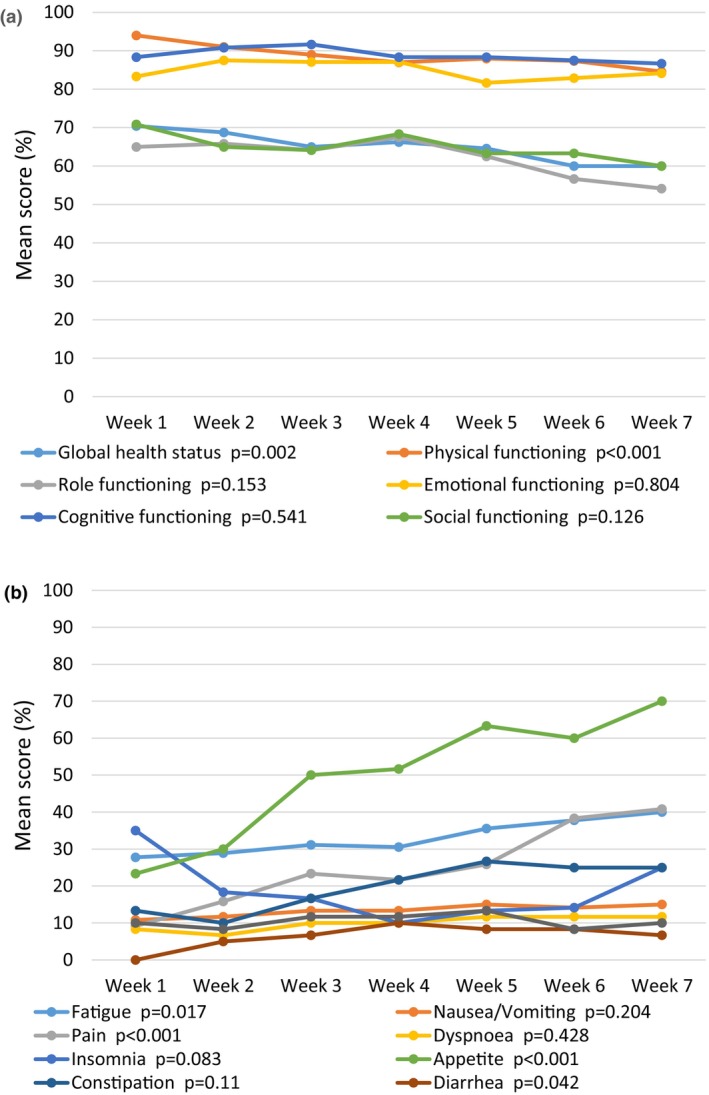
Patient‐reported quality of life from week 1 to week 7. (a) QLQ‐C30 functional scales and global health status. (b) QLQ‐H&N43 symptom scales. Tests for statistical significance were performed for change from first to last measurement with paired‐sample *T*‐test, double‐sided.

**Figure 5:**
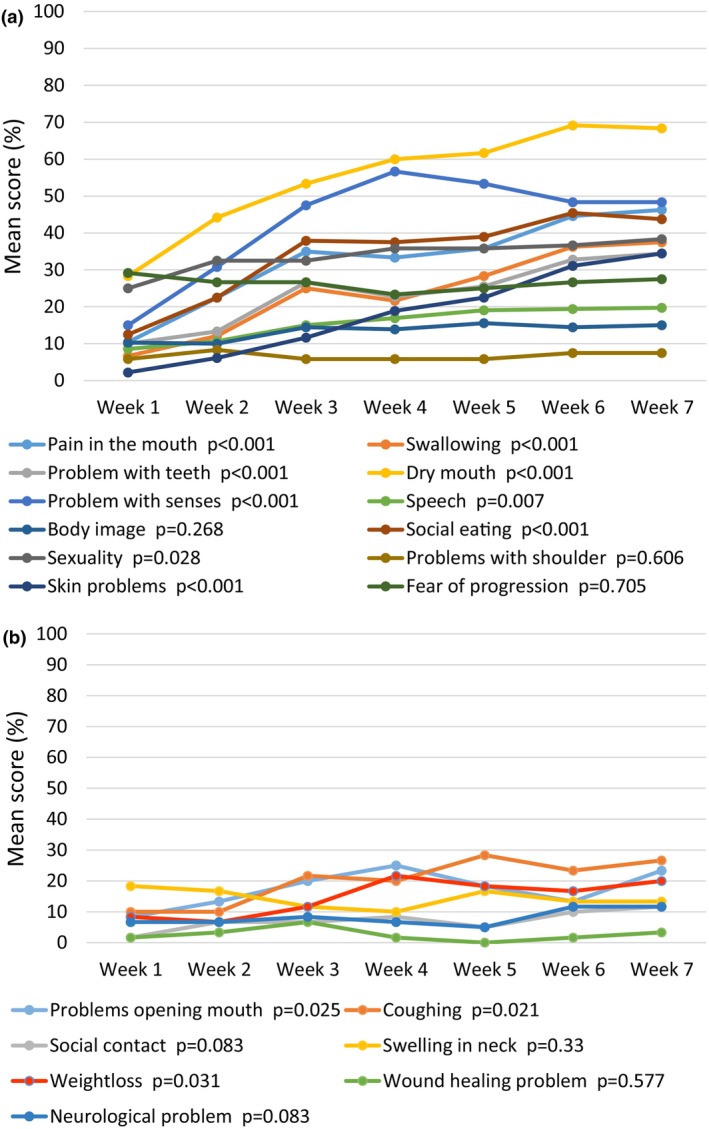
Patient‐reported quality of life from week 1 to week 7. (a) QLQ‐H&N43 multi‐item scales. (b) QLQ‐H&N43 single‐item scales. Tests for statistical significance were performed for change from first to last measurement with paired‐sample *T*‐test, double‐sided.

The results of the quality‐of‐life instruments QLQ‐C30 and QLQ‐H&N43 are shown in Figures 4 and 5, respectively. Global health status and physical functioning showed a statistically significant decline from the first to the last measurement. As seen in the figures, several symptom scales, particularly those related to symptoms from the oral cavity, throat or eating, also showed significant changes over time. The increase in neurological symptoms in the QLQ‐H&N43 was attributed to participants undergoing concomitant chemotherapy.

### Participant preference

Before the final week, participants freely chose which mask to use during the last four fractions based on their preferences and experiences. The outcome was that twelve (60%) chose the open mask and eight (40%) chose the closed mask. This result did not show a statistically significant difference from a situation where both masks were preferred equally, that is, 50% for each (*P* = 0.503, one‐sample binomial test).

## Discussion

In this study, participants found that the open mask felt less confining, and for some, it reduced feelings of claustrophobia compared to the closed mask. The open mask also provided better opportunities for interaction with the environment. On the other hand, the closed mask made it easier to achieve the correct treatment position, making the setup process smoother and faster for both patients and staff. Participants also perceived the closed mask as more stable. Overall, most participants did not have a strong preference for either mask.

The fixation mask could induce negative feelings of confinement and claustrophobia, as shown to be an issue for up to 26% of patients,^2^, ^6^, ^8^ but it could also evoke positive feelings of being able to remain still. This is consistent with previous studies demonstrating that patients can have both positive and negative feelings towards the mask.^5^, ^10^ Although many patients find the mask fixation itself unpleasant, concerns about a lack of fixation leading to poorer treatment outcomes can also be troubling, and firm and stable fixation was generally seen as beneficial. As in previous studies, support from staff was important for patients to feel secure and cope with treatment.^5^, ^9^

The HADS anxiety subscale did not detect significant differences in anxiety scores between weeks with open and closed masks, but it did show a significant reduction in anxiety over time, consistent with published results.^7^ This aligns with participants' interview responses, which describe how it gradually became easier to handle being immobilised in the mask.

The HADS depression subscale results showed large individual variations, with minor changes over time, suggesting that mood primarily depends on person‐related factors rather than treatment‐related factors. Mood variations among participants were also noted in the interviews.

There are no other studies, to our knowledge, where participants alternated between open and closed masks, but studies comparing groups have been conducted. A study by Mulla et al.^12^ randomised 40 participants to either an open or closed mask and reported better overall satisfaction and a trend towards less anxiety, tightness and pain with the open mask. Another study by Wiant et al.^15^ with 50 participants randomised to either an open or closed mask reported a non‐significant trend towards lower anxiety and claustrophobia with the open mask.

The participants' choice of mask for the last four treatments provides a good measure of their preference regarding the mask, as the choice also had direct consequences for the participant. When given the option, a slight majority (60%) chose the open mask. This was not statistically significantly different from 50% meaning that we cannot say whether one of the masks was preferred more than the other. This indicates that, despite the fact that the open mask was perceived as less confined and claustrophobic, there was no strong preference for either mask among the participants. The interviews confirmed that many participants had no strong preference and saw pros and cons with both masks. This suggests that factors such as quick and easy positioning and good support for lying still might be as important for the patient's experience as the comfort in the mask. However, one participant tolerated only the open mask due to anxiety, and although all other participants could undergo treatment with either mask, interviews revealed that some participants had to put more effort into mental preparation or alternatively, used sedative medication when using the closed mask. This aligns with findings in a study by Li et al.^13^, showing that open masks perform well for patients with mild or moderate claustrophobia.

A prospective study by Clover et al.^8^ suggested predictors for increased risk of anxiety among patients receiving head and neck radiotherapy. These predictors include the use of psychotropic drugs, fear of confinement, fear of having the face covered, fear of being restrained and a history of anxiety attacks. These predictors could potentially guide the selection of patients particularly suitable for the open mask, though further validation is needed.

One participant dropped out due to swelling, making the closed mask fit poorly. In clinical practice, an open mask is not readily available, and this type of situation must be solved by cutting away parts of the mask. This leads to a less stable mask, and there have been concerns that patient positioning may be degraded by such a measure. The open masks studied here have reinforcements, ensuring stability remains good even though the mask does not cover as much of the head. The stability and accuracy of positioning with the open mask compared to the closed one is being studied in a sub‐study of this project, and the results will be published separately.

The development of symptom scales and function scales QLQ‐C30 and QLQ‐H&N43 followed what could be expected from previously published studies,^22^ with increasing symptoms from the mouth and throat after 2–3 weeks of treatment. Simultaneously, we observed a decline in functions related to eating and appetite. A limitation in this measurement is that baseline data were missing, so we could only track development starting after 1 week of treatment.

The HADS is well‐validated for screening or repeated use as frequently as weekly, to measure the impact of an intervention over time.^18^ A statistically significant difference in anxiety scores could have indicated a measurable difference in perceived anxiety between the two masks, but no such difference was found. The clear decrease in anxiety scores over the course of treatment suggests that starting radiation treatment as such, rather than the type of mask that is used, causes anxiety, which subsides as patients become accustomed to the treatment situation.

A strength of this study was that participants could directly compare the two mask types, providing a clear understanding of the differences in their experiences and which mask they preferred. By combining quantitative methods with quality‐of‐life measurements and participants' choice of mask with qualitative methods using semi‐structured interviews, we quantified participants' experiences with the two mask types and gained insight into the reasons behind their preferences. This enhanced our ability to make recommendations despite the limited study sample.

A limitation of this study was the limited number of participants, which reduced the ability to draw statistically reliable conclusions from the quantitative data from HADS and QoL instruments. Another limitation was that participants' conditions changed throughout treatment due to increasing side effects, which may make it difficult for them to consistently and fairly compare the masks. We attempted to counteract this by randomising the starting mask, but it may still have influenced the results. Despite these limitations, we believe the conclusion from this mixed‐methods study is that patients receiving radiotherapy should be offered a choice between open and closed masks. However, further research is needed to understand mask‐related anxiety better. As this study showed that mask type preference varies among patients, more research is needed to develop guidelines on how to recommend the most appropriate mask for each individual. Further research is also needed to validate that the two mask types provide sufficient precision and stability for patient positioning. This question is being addressed in another sub‐study of this research project, with results to be published later.

## Conclusion

For the majority of patients, both open and closed masks are useful options, and although an open mask is often preferred, other factors can be considered when deciding which mask to use. For patients who are not able to tolerate a closed mask, the option of an open mask is a safe, well‐tolerated alternative.

## Declaration of generative AI in scientific language

In the preparation of this text, ChatGPT has been used to improve grammar and English language.

## Funding Information

Örebro Research Committee Grant Number OLL‐942106.

## Conflict of Interest

The authors have no conflict of interest in this work.

## Ethics Approval

The study received approval from the Swedish Ethics Review Board (D.nr 2019‐00249).

## Data Availability

The data that support the findings of this study are available from the corresponding author upon reasonable request.
